# Internal Exposure to BTEX in Tropical Children: Does Exposure Speed Up Pubertal Development?

**DOI:** 10.3390/antiox14101164

**Published:** 2025-09-25

**Authors:** Yao Lu, Qin Zhou, Dan Wang, Yu-Ling Luan, Ying Guo

**Affiliations:** 1 Guangdong Key Laboratory of Environmental Pollution and Health, School of Environment and Climate, Jinan University, Guangzhou 511443, China; ly0907@stu2022.jnu.edu.cn (Y.L.); cuilan@stu2022.jnu.edu.cn (Q.Z.); phd1021@stu2020.jnu.cn (Y.-L.L.); 2 Hainan Branch, Shanghai Children’s Medical Center, School of Medicine, Shanghai Jiao Tong University, Sanya 572000, China

**Keywords:** BTEX, internal exposure, precocious puberty, early puberty, oxidative DNA damage

## Abstract

Benzene, toluene, ethylbenzene, and xylene (BTEX) are a common class of volatile organic compounds linked to adverse health outcomes. Although BTEX have been shown to have endocrine disrupting properties, their potential impacts on pubertal development in children remain unclear. In this study, for the first time, we investigated the possible association of BTEX exposure with precocious puberty (PP) and early puberty (EP) in children. We conducted a case–control study that included 246 children diagnosed with PP or EP and a controls category matched for sex and age. Urinary concentrations of seven BTEX metabolites and the oxidative DNA damage biomarker 8-hydroxy-2′-deoxyguanosine (8-OHdG) were measured by high-performance liquid chromatography-tandem mass spectrometry. Compared with the control groups, urinary catechol (1,2-DB) levels were significantly higher in both PP (median: 85.3 vs. 42.4 μg/g creatinine) and EP children (median: 62.0 vs. 48.1 μg/g creatinine). Binary logistic regression models showed that 1,2-DB was positively associated with EP (OR = 1.36, 95% CI: 1.10, 1.69), while *trans*,*trans*-muconic acid (MU) was negatively correlated with PP (OR = 0.47, 95% CI: 0.24, 0.92), and PGA was negatively correlated with EP (OR = 0.73, 95% CI: 0.56, 0.93) adjusted for confounders. Stratified analyses showed that the relationship between BTEX metabolites and EP varied by parental education level. Our findings revealed that exposure to BTEX, especially benzene, may influence pubertal timing in children, but there was no relationship between oxidative DNA damage and PP or EP. The biological mechanism of BTEX exposure affecting pubertal development requires further investigation.

## 1. Introduction

Volatile organic compounds (VOCs) are ubiquitous in the environment and originate from various sources, such as paints, industrial solvents, biomass combustion, automobile exhaust, tobacco smoke, and office printers [1]. Benzene, toluene, ethylbenzene, and xylene (BTEX) are the most abundant VOCs, accounting for more than 60% of VOCs levels in urban areas [2]. Humans are exposed to BTEX through three primary pathways: inhalation, dermal contact, and ingestion [3]. Exposure to BTEX may cause adverse health effects on nervous system, respiratory system, cardiovascular system and even links to cancer [4,5,6]. Some of these health outcomes involved the key mechanism of oxidative stress, such as oxidative DNA damage [7,8]. Exposure to BTEX was also associated with endocrine disruption. BTEX has been known to have endocrine disrupting properties [9]. Benzene may interfere with pituitary hypothalamus adrenocortical action and reduce thyroid stimulating hormone levels [10]. Exposure to BTEX has also been associated with sex hormone disruption, which may further interfere with the function of reproductive system. It was reported that occupational exposure to toluene was linked to reductions in hormones like luteinizing hormone, follicle stimulating hormone (FSH), and testosterone [11]. Some studies reported the correlations between benzene exposure and alterations in sex hormone levels in American males and females [12,13]. Children are more sensitive to environmental pollutants as children have immature metabolic pathways [14]. Childhood exposure to BTEX may potentially have negative impacts on children’s growth and development. Some evidences have showed that exposure to endocrine disrupting chemicals (EDCs) may cause the increase of precocious puberty (PP) [15].

Precocious puberty is an endocrine disorder of abnormal growth and development in children, defined as the occurrence of secondary sexual characteristics before the age of 8 years for girls or 9 years for boys [16]. Pubertal development that begins earlier than normal development age but not within the age definition of PP is defined as early puberty (EP) [17]. Current studies have mainly focused on the PP, with fewer studies on the EP. In recent years, the prevalence of PP is increasing and occurs mostly in girls [18]. It was reported that the prevalence of PP in children was between 1/5000 and 1/10,000 [17]. An epidemiologic study in South Korea reported an overall prevalence of central precocious puberty (CPP) of 193.2 per 100,000 children from 2008 to 2014, with 10.9/100,000 in boys and 410.6/100,000 in girls [19]. A survey based on the school population in 2019 in Guangdong Province, China, showed a prevalence of PP of 6.19% among 4058 students in grades 1–3, with a prevalence of 11.47% among girls and 3.26% among boys [20].

Precocious puberty can accelerate physical development in children and lead to premature epiphyseal closure, resulting in short height in adulthood. It can also lead to psychological problems in children, as well as increase risk of diabetes, infertility, breast cancer, and other diseases in adulthood [21]. There are many factors that contribute to PP, including genetic factors, dietary habits, environmental factors, and psychosocial stress [22]. With the development of industry, the impact of environmental contaminants, particularly EDCs, on PP has become increasingly prominent. Recent studies have reported that exposure to phthalates, bisphenol A, and anabolic steroid hormones may affect pubertal timing [18,23,24]. In addition, it was found that mixed exposure to organic UV filters and phthalates were related to earlier pubertal development in girls and delayed pubertal onset in boys [25]. A case-crossover study in China suggested that short-term exposure to PM_2.5_ and PM_10_ may increase PP risk [21]. Nevertheless, research on the relationship between BTEX exposure and PP or EP in children remains limited.

Due to the high volatility of BTEX, the concentrations of BTEX in the ambient air may not accurately reflect individual’s exposure. Urinary BTEX metabolites can serve as common biomarkers of BTEX exposure, which has the advantage of non-invasive sampling of urine and longer physiological half-lives [26]. In this study, we determined seven BTEX metabolites concentrations in children’s urine samples and explored the possible associations of BTEX exposure with PP and EP. Concentrations of 8-hydroxy-2′-deoxyguanosine (8-OHdG) in urine were also measured to evaluate oxidative DNA damage impact on PP and EP. This study is the first to investigate potential relationship between BTEX exposure and pubertal development.

## 2. Materials and Methods

### 2.1. Population Recruitment and Sample Collection

We recruited 246 children aged 6–10 years diagnosed with PP or EP from six elementary school in Sanya City, Hainan Province, China, between November and December 2022. Children in the normal development group from the same study area were category matched by sex and age with the case group to serve as control groups. All children participating in this study signed an informed consent and completed a questionnaire with parent’s help. The questionnaire included age, height, weight, body mass index (BMI), birth weight, mode of delivery, whether or not the only child, breastfeeding status, girls’ experience of menarche, past medical history (e.g., neonatal jaundice, meningitis, etc.), sleep time, dietary intake, mother’s menarche age, maternal gestational age, mother’s unfavorable factors during pregnancy, parental education level, monthly household income, experience of severe traumatic events, whether or not they had accidentally consumed birth control pills, hormonal drug use, adult makeup use, vitamin D intake, and living room environment. First morning urine samples were collected from each child in 12 mL polypropylene tubes and delivered to the laboratory where they were stored at −20 °C prior to analysis. After excluding lost urine samples, a total of 473 urine samples were ultimately included in the analysis, from 61 PP children, 185 EP children, and 227 corresponding control children. The study was approved by the Ethics Committee of Jinan University, China (JNUKY-2022-006).

### 2.2. Physical Examination and Assessment of Puberty Status

Stages of pubertal development were evaluated according to Tanner stages [27,28]. Physical examination of children was assessed by specialized pediatricians through inspection and palpation. The breast development of girls was assessed through inspection and palpation and was categorized into B1–B5. The testicular volume (TV) of boys was also estimated by palpation and was categorized into four grades (T1–T4). Pubic hair stage was assessed by inspection and categorized into five growth stages from PH1 to PH5. The diagnosis criteria of precocious puberty were determined in accordance with the Diagnosis and Treatment Guidelines for Precocious Puberty issued by Chinese Ministry of Health in 2010. Girls were diagnosed with PP if breast development was at B2 before age 8 years, and boys were diagnosed with PP if testicular volume reached T2 (4 ≤ TV < 12 mL) before age 9 years. The control group of PP were girls with normal development before 8 years and boys with normal development before 9 years. The inclusion criteria for early puberty: Pubertal development that begins earlier than normal development age but not within the age definition of PP [17]. Early puberty was diagnosed if breasts development reached B2 between the ages of 8 and 10 years for girls and TV reached T2 between the ages of 9 and 10 years for boys. Girls with normal development the ages of 8 and 10 years and boys with normal development the ages of 9 and 10 years were regarded as the controls of EP.

### 2.3. Chemicals and Reagents

Seven native standards of BTEX metabolites were used, including benzene metabolites S-phenyl mercapturic acid (PMA), 1,2-dihydroxybenzene (catechol, abbreviation: 1,2-DB), *trans*,*trans*-muconic acid (MU), toluene metabolite N-Acetyl-S-(benzyl)-L-cysteine (BMA), ethylbenzene metabolite phenylglyoxylic acid (PGA), o-xylene metabolite 2-methyl hippuric acid (2MHA), m-xylene metabolite 3-methyl hippuric acid (3MHA). 8-OHdG was used as the biomarker of oxidative DNA damage. Creatinine was used for analyte concentration correction. The internal standard PMA-d_5_, 1,2-DB-d_4_, MU-d_4_, BMA-d_5_, PGA-d_5_, 2MHA-d_7_, 3MHA-d_7_, ^15^N_5_-8-OHdG, and creatinine-d_3_ were used to spiked the samples to quantify concentrations. The purities of native standards and internal standards were all over 95%. The details of native standards and their internal standards are shown in Table S1. HPLC-grade methanol, water, acetic acid, formic acid, and ammonium acetate were purchased from Fisher Scientific (Waltham, MA, USA). β-glucuronidase enzyme was purchased from Sigma-Aldrich (≥100,000 units/mL, St. Louis, MO, USA).

### 2.4. Sample Preparation and Instrumental Analysis

Direct dilution method was used for urinary BTEX metabolites preparation. Briefly, urine sample was thawed at room temperature and vortexed for thorough mixing. A 200 μL aliquot of urine sample was mixed with 200 μL of β-glucuronidase enzyme (diluted to 500 units/mL with 1 M ammonium acetate solution) in the 1.5 mL centrifuge tube and incubated in the shaker overnight (37 °C, 150 rpm). Subsequently, the mixture was vortexed and then centrifuged (12,000 rpm, 5 min) and the supernatant was filtered through a 0.22 μm polyether sulfone membrane. Then, 100 μL of the mixed solution after membrane filtration was taken into the LC vial, spiked with 50 μL of internal standard solution (100 ng/mL), and diluted with 2% aqueous acetic acid (*v*/*v*) to 0.5 mL for further analysis.

High-performance liquid chromatography (ExionLC) coupled with an AB SCIEX 6500 electrospray ionization triple-quadrupole mass spectrometer (ESI-MS/MS) was employed to analyze all the samples. For BTEX metabolites, chromatographic separations were performed using a BETASIL C18 column (100 mm × 2.1 mm × 5 μm; Thermo Fisher Scientific, Waltham, MA, USA) at 40 °C. The mobile phases were 0.1% aqueous acetic acid (*v*/*v*) (A) and methanol (B) at a flow rate of 0.4 mL/min, and the sample injection volume was 5 μL (Table S2). Negative ionization mode and multiple reaction monitoring mode were used to determine concentrations of BTEX metabolites. Detailed mass spectrometry parameters are shown in Table S3. Urinary 8-OHdG and creatinine were diluted with water in 1:20 and 1:10,000 respectively for sample preparation. The details of sample preparation and determination methods of 8-OHdG and creatinine are shown in Text S1.

### 2.5. Quality Assurance and Quality Control

Three procedural blanks, three blank spiked samples, and three matrix spiked samples were included in each batch of analysis. For analytes with background concentrations, the measured concentration of BTEX metabolites in the urine sample was subtracted from the background concentrations to obtain the true concentrations. The average recoveries of matrix spiked samples were in the range of 88–132% and average recoveries of internal standards of all samples were in the range of 61–114% (Table S4). The reproducibility of the method was assessed by taking 4–5% of urine samples from each batch, and the relative standard deviation (RSD) of most parallel samples was less than 20%. In instrumental analysis, a methanol shot was analyzed after every 15 samples to detect target compound residues and prevent cross-contamination, while a midpoint calibration concentration was injected to check the instrument’s stability. For all analytes, the calibration curve regression coefficients were greater than 0.995. The limits of detection (LODs) of method were calculated based on the 3-fold signal-to-noise ratio (S/N) and varied from 0.01 to 0.49 ng/mL.

### 2.6. Statistical Analysis

Concentrations of analytes below LODs were replaced by LODs/√2. Concentrations of BTEX metabolites and 8-OHdG were adjusted by the corresponding creatinine concentrations and reported as μg/g creatinine. The Kolmogorov–Smirnov normality test combined with Q-Q plot were used to examine the distribution of data normality, showing that concentrations of all analytes were right-skewed. Exposure profiles were described by detection frequency (DF), geometric mean (GM), and median. The Mann–Whitney U test and chi-square test were performed to assess the differences in continuous and categorical variables between the case and control groups, respectively. Confounding variables were identified based on the comparison results. Spearman correlation analysis was employed to evaluate the correlation among each target analyte. Before regression analyses, BTEX metabolites and 8-OHdG concentrations were ln-transformed. The associations between BTEX metabolites and 8-OHdG in PP and EP children were evaluated by multiple linear regression model, adjusted for sex, age, BMI, mode of delivery, whether or not the only child, and parental education level. A binary logistic regression model was conducted to estimate the association of BTEX metabolites and 8-OHdG with PP and EP in children without adjustment or with adjusting for BMI, mode of delivery, whether or not the only child, and parental education level to calculate the odds ratio (OR) and 95% confidence interval (CI). Weighted quantile sum (WQS) regression was used to analyze the joint effect of BTEX metabolite mixture on 8-OHdG, PP, and EP and calculate the weight of each BTEX metabolite adjusted for the confounders mentioned previously. The WQS model divided the dataset into the training set (40%) and the validation set (60%), set the joint effect direction to be either positive or negative, and performed a bootstrap procedure 1000 times to derive the weights (0–1) and test the significance of the mixture [29]. Stratified analysis was conducted according to sex and parental education level adjusted for confounders.

Statistical analyses were performed with SPSS (version 25.0, IBM, Armonk, NY, USA) and R (version 4.4.1), while visualization of the data was performed using Origin 2025 (OriginLab Corporation, Northampton, MA, USA) and GraphPad Prism (version 8.0.2, San Diego, CA, USA). A two-tailed *p*-value of less than 0.05 was considered statistically significant.

## 3. Results

### 3.1. Demographic Characteristics of the Participants

Four hundred and seventy-three children were divided into PP or non-precocious puberty (non-PP) subgroups (n = 109) and EP or non-early puberty (non-EP) subgroups (n = 364) (Table 1). Among them, 13 boys (21.3%) and 48 girls (78.7%) were diagnosed with PP. The average ages of the PP and non-PP children were 6.89 ± 0.61 years and 6.92 ± 0.61 years, respectively. The PP children showed significantly higher BMI values than the non-PP children (*p* < 0.01). In addition, significant differences were found between PP and non-PP children in terms of mode of delivery, whether or not the only child, and level of parental education (*p* < 0.05 or *p* < 0.01). Consumption frequencies of vegetables and fruits were also significantly different between PP and non-PP children (Table S5).

A total of 185 children were diagnosed with EP, containing 11 boys (5.9%) and 174 girls (94.1%). The mean ages of the EP and non-EP children were 8.36 ± 0.49 years and 8.39 ± 0.50 years, respectively. Compared with the non-EP children, the EP children had significantly higher BMI (*p* < 0.01). Moreover, a higher percentage of EP children were the only child compared with non-EP children (*p* < 0.01). The EP children and non-EP children exhibited similar dietary frequency patterns. However, the non-EP children had a higher frequency of vitamin D intake than EP children (*p* < 0.01).

### 3.2. Urinary Occurrence of BTEX Metabolites and 8-OHdG in Children

**Figure 1:**
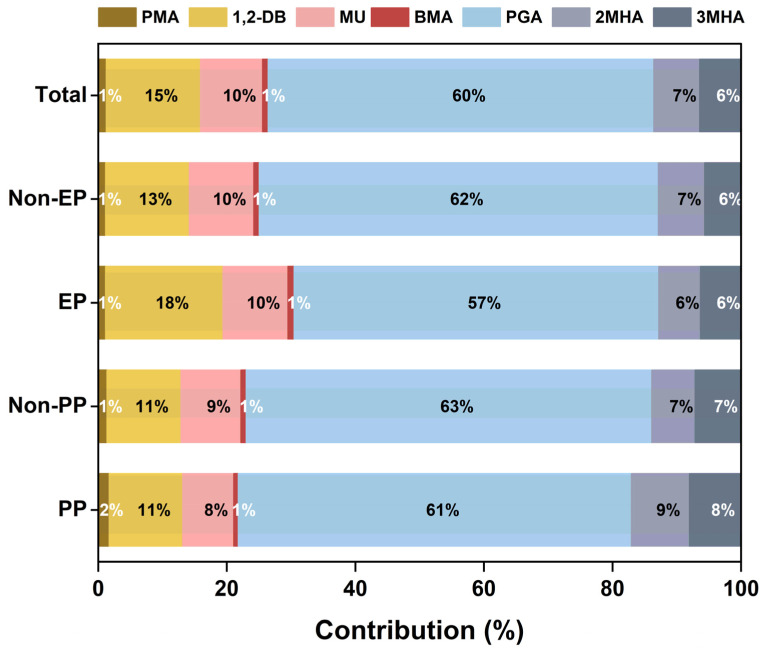
Composition profiles of creatinine-adjusted BTEX metabolites concentrations (μg/g creatinine) in urine samples from all children or different subgroups of children. PP, precocious puberty; EP: early puberty.

The creatinine-adjusted and unadjusted concentrations of BTEX metabolites and 8-OHdG in children’s urine are presented in Table 2, Table 3 and Table S6, respectively. All analytes were detected in children’s urine samples (DFs ≥ 87.5%). Among the seven BTEX metabolites, PGA had the highest percentage of total concentrations, accounting for 60%, followed by 1,2-DB and MU (Figure 1). As shown in Table 2, the median concentrations of urinary BTEX metabolites varied from 3.74 μg/g creatinine (BMA) to 496 μg/g creatinine (PGA) in PP children. In comparison to non-PP children, PP children had significantly higher concentrations of 1,2-DB (median: 85.3 vs. 42.4 μg/g creatinine) and lower concentrations of MU (median: 47.4 vs. 60.0 μg/g creatinine) (*p* < 0.05). The median concentration of 1,2-DB in PP girls was also higher than that in non-PP girls (*p* < 0.05). In addition, the median values of urinary BTEX metabolites ranged from 4.60 μg/g creatinine (BMA) to 348 μg/g creatinine (PGA) in EP children (Table 3). Compared with non-EP children, EP children had significantly higher levels of 1,2-DB (median: 62.0 vs. 48.1 μg/g creatinine) and lower levels of PGA (median: 348 vs. 462 μg/g creatinine) (*p* < 0.01). Similar concentration differences were observed in the EP girls and non-EP girls. Moreover, urinary levels of PGA were significantly elevated in the PP children compared to the EP children (*p* < 0.05) (Table S7). The median values of 8-OHdG in PP and EP children were 5.31 μg/g creatinine and 5.58 μg/g creatinine, respectively. Furthermore, no statistically significant variation in urinary 8-OHdG levels was observed between the case and control groups or between PP and EP children.

### 3.3. Relationship of 8-OHdG with BTEX Metabolites

**Figure 2:**
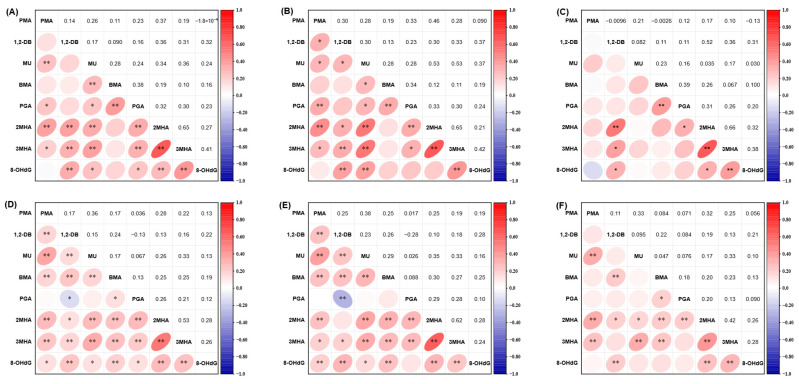
Spearman correlation analysis between BTEX metabolites and 8-OHdG of precocious puberty and non-precocious puberty subgroup children (**A**), precocious puberty children (**B**), non-precocious puberty children (**C**), early puberty and non-early puberty subgroup children (**D**), early puberty children (**E**), and non-early puberty children (**F**). *: *p* < 0.05. **: *p* < 0.01.

Correlations were examined between BTEX metabolites and 8-OHdG by Spearman correlation analysis (Figure 2). In PP children, 1,2-DB, MU, and 3MHA were correlated with 8-OHdG, with Spearman’s correlation coefficients (*r* value) varying from 0.37 to 0.42 (Figure 2B), while three metabolites 1,2-DB, 2MHA, and 3MHA were correlated with 8-OHdG in non-PP children (*r* = 0.31–0.38) (Figure 2C). Similar, common correlations were found between BTEX metabolites and 8-OHdG in EP children (*r* = 0.16–0.28), except for PGA (Figure 2E). Only 1,2-DB, 2MHA, and 3MHA were correlated with 8-OHdG in non-EP children (*r* = 0.21–0.28) (Figure 2F).

**Figure 3:**
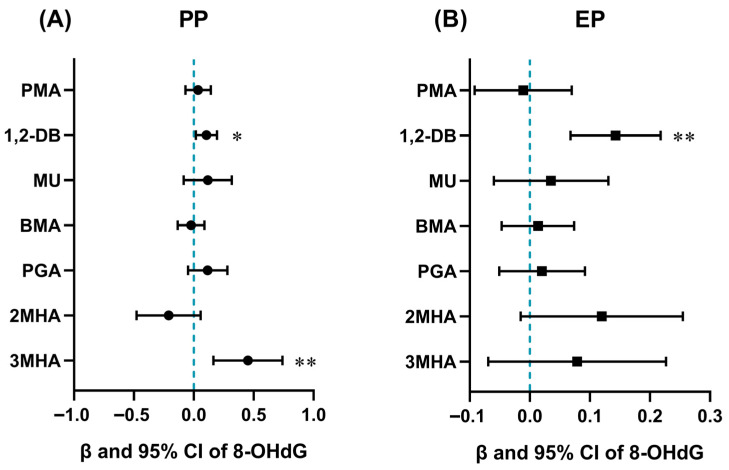
Association of urinary BTEX metabolites and 8-OHdG in precocious puberty (PP) children (**A**) and early puberty (EP) children (**B**) using multiple linear regression model adjusted for sex, age, body mass index, mode of delivery, whether or not only child, and parental education level. Levels of BTEX metabolites and 8-OHdG were ln-transformed before analyses. 8-OHdG: 8-hydroxy-2′-deoxyguanosine. *: *p* < 0.05. **: *p* < 0.01.

The relationships between BTEX metabolites and 8-OHdG levels in children were also analyzed by multiple linear regression models. Following confounder adjustment, 1,2-DB (*β* = 0.11, 95% CI: 0.02, 0.19) and 3MHA (*β* = 0.45, 95% CI: 0.16, 0.74) showed significant correlation with the elevation of 8-OHdG in the PP children (*p* < 0.05) (Table S8 and Figure 3A), while 1,2-DB showed significantly positive association with 8-OHdG in the EP children (*β* = 0.14, 95% CI: 0.07, 0.22, *p* < 0.01) (Figure 3B).

**Figure 4:**
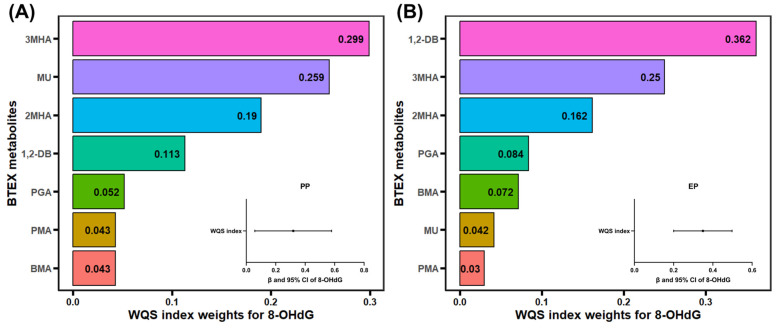
WQS regression model. Estimated weights of BTEX metabolite mixture associated with 8-OHdG in precocious puberty (PP) children (**A**), and early puberty (EP) children (**B**) based on ln-transformed urinary creatinine-adjusted concentrations. Sex, age, body mass index, mode of delivery, whether or not only child, and parental education level were adjusted in models. 8-OHdG: 8-hydroxy-2′-deoxyguanosine; WQS, weighted quantile sum.

The WQS model was used to assess the joint effect of the BTEX metabolite mixture on 8-OHdG. In the PP children, the WQS index of the BTEX mixture exhibited significant correlation with 8-OHdG (Figure 4A, *β* = 0.32, 95% CI: 0.06, 0.58, *p* < 0.05), with the highest weight for 3MHA (0.299). The WQS index also demonstrated a significant association with elevated levels of 8-OHdG in the EP children (Figure 4B, *β* = 0.35, 95% CI: 0.20, 0.50, *p* < 0.01), with 1,2-DB (0.362) contributing the highest weight.

### 3.4. Association of BTEX Metabolites and Oxidative DNA Damage with Precocious Puberty

**Figure 5:**
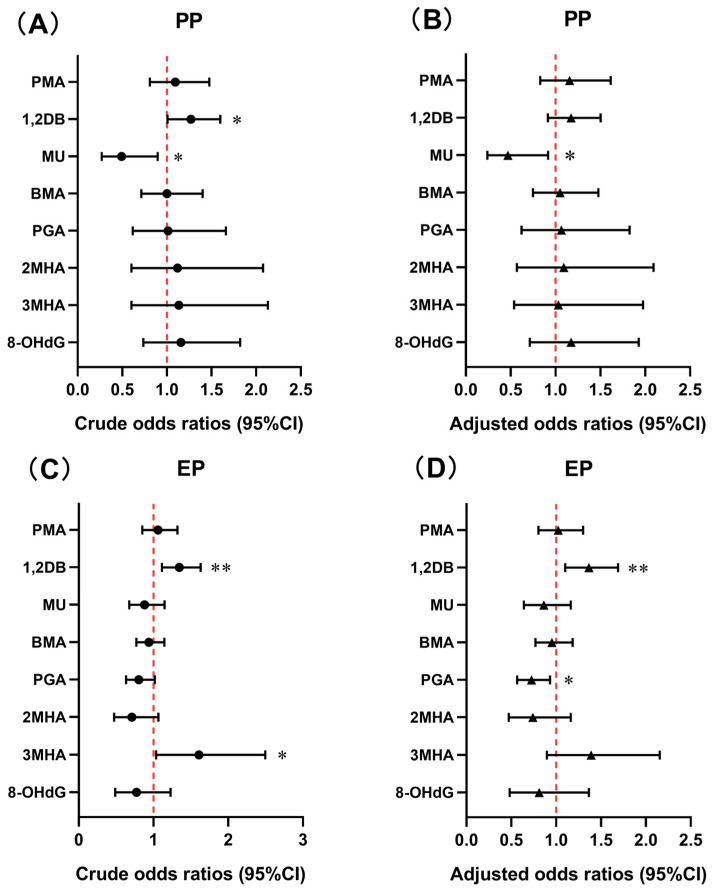
Associations of urinary BTEX metabolites and 8-OHdG with precocious puberty (PP) and early puberty (EP) using binary logistic regression models. (**A**) Crude odds ratios and 95%CI for PP; (**B**) Adjusted odds ratios and 95%CI of PP adjusted for body mass index, mode of delivery, whether or not only child, and parental education level. (**C**) Crude odds ratios and 95%CI for EP; (**D**) Adjusted odds ratios and 95%CI of EP adjusted for body mass index, mode of delivery, whether or not only child, and parental education level. Levels of BTEX metabolites and 8-OHdG were ln-transformed before analyses. *: *p* < 0.05. **: *p* < 0.01.

In the binary logistic regression models, 1,2-DB had a significantly positive association with PP (OR = 1.27, 95% CI: 1.01, 1.60, *p* < 0.05), while MU had a significantly negative association with PP (OR = 0.49, 95% CI: 0.27, 0.90, *p* < 0.05) in the crude model (Figure 5A and Table S9). After adjustment for BMI, mode of delivery, whether or not the only child, and parental education level, there was no association between 1,2-DB and PP. Only MU was still significantly and negatively associated with PP (OR = 0.47, 95% CI: 0.24, 0.92, *p* < 0.05) (Figure 5B).

There was no correlation between urinary 8-OHdG levels and PP.

The effects of BTEX metabolite mixture on PP were assessed by the WQS regression models. The WQS index of BTEX metabolite mixture showed no association with PP adjusted for confounders (Table S10).

Due to the small number of boys, the sex stratification analysis only focused on the girls. When boys were excluded and the girl population was analyzed, 1,2-DB (OR = 1.32, 95% CI: 1.02, 1.70) and 3MHA (OR = 3.06, 95% CI: 1.02, 9.15) levels showed positive association with PP in girls and MU (OR = 0.42, 95% CI: 0.20, 0.88) showed negative association with PP in girls in the crude model (*p* < 0.05) (Table S11). However, there was no association of BTEX metabolites with PP after adjustment for confounders.

### 3.5. Association of BTEX Metabolites and Oxidative DNA Damage with Early Puberty

In the binary logistic regression models, increased urinary 1,2-DB and 3MHA levels were significantly correlated with elevated OR for EP before adjustment for confounders (OR = 1.35, 95% CI: 1.11, 1.63, *p* < 0.01; OR = 1.61, 95% CI: 1.04, 2.50, *p* < 0.05) (Figure 5C and Table S12). After adjustment for confounders, 1,2-DB was still significantly and positively associated with EP (OR = 1.36, 95% CI: 1.10, 1.69, *p* < 0.01), whereas PGA was significantly and negatively associated with the OR for EP (OR = 0.73, 95% CI: 0.56, 0.93, *p* < 0.05) (Figure 5D).

No association of 8-OHdG with EP was observed in both crude and adjusted models.

In the WQS model, the WQS index did not have association with EP with adjustment for confounders in the positive direction (Table S10). When the effect was set in the negative direction, the WQS index was significantly associated with EP (OR = 0.51, 95% CI: 0.34, 0.76), with PGA having the highest weight of 0.412.

Similarly, when boys were excluded, 1,2-DB and 3MHA were positively associated with EP in girls in the crude model, whereas 1,2-DB was positively related to EP (OR = 1.34, 95% CI: 1.08, 1.66, *p* < 0.01) and PGA was negatively related to EP (OR = 0.74, 95% CI: 0.57, 0.96, *p* < 0.05) in girls adjusted for confounders (Table S13). When stratifying by parental education level, PMA was significantly positively associated with EP when both parents had less than high school education levels adjusted for confounders (OR = 3.66, 95% CI: 1.32, 10.2, *p* < 0.05) (Table 4). When at least one of the parents’ education levels was high school or above, significantly positive association was found between 1,2-DB and EP (OR = 1.35, 95% CI: 1.06, 1.74), while PGA showed significantly negative association with EP (OR = 0.74 95% CI: 0.56, 0.97) (*p* < 0.05). The results suggested that the association between BTEX metabolites and EP varied by parental education level.

## 4. Discussion

Our study determined urinary levels of seven BTEX metabolites to assess the relationship of BTEX exposure with PP and EP in children from Sanya. The results suggested that school children were widely exposed to BTEX. Urinary 1,2-DB concentrations were significantly higher in PP and EP children than in their corresponding controls, which indicated that PP and EP children may have been exposed to higher levels of benzene. Logistic regression revealed that 1,2-DB was positively associated with PP and EP. We also found a negative association of MU with PP and PGA with EP. Stratified analysis further indicated that BTEX exposure had a greater impact on EP in girls and the association between 1,2-DB and EP was more significant in children from families with parental education levels were high school or above. These results suggest that BTEX exposure, especially benzene exposure, is associated with PP and EP in children. In addition, no association was found between 8-OHdG and PP or EP, suggesting that the effect of BTEX on PP and EP was not associated with oxidative DNA damage though several benzene and xylene metabolites showed positive correlation with 8-OHdG in PP and EP children.

Urinary levels of BTEX metabolites have been determined in different populations, including children, adults, and pregnant women [3,30,31,32,33,34,35,36,37,38,39,40,41] (Table S14). The levels of some BTEX metabolites were higher in this study compared to others. The GM levels of PGA and 2MHA in the urine of PP children were 1.8 and 1.6 times higher than children aged 6–11 years from the USA [32]. When compared to 6–12-year-old children in Guangzhou, China, the unadjusted urinary GM levels of PMA, MU, and 2MHA in PP children were 74.2, 3.1, and 3.9 times higher, respectively [35]. Compared with children in South China, the GM levels of PMA and 1,2-DB were 57.2 and 8.1 times higher in the PP children and 46.3 and 10.1 times higher in the EP children [38]. In our study, children with PP and EP may be exposed to elevated levels of benzene, ethylbenzene, and o-xylene, which are widely sourced from automobile exhaust, tobacco smoke, indoor building materials, organic solvents, personal care products, etc. [31]. Benzene and ethylbenzene are also used in children’s stationery, and ethylbenzene even exists in products of children and toys [42]. Children are likely to spend more time indoors and are widely exposed to indoor BTEX. Previous studies showed that the total concentration of BTEX in the air of Guangzhou in South China (610 μg/m^3^) was higher than that in other cities and regions in China, such as Beijing (18.5 μg/m^3^) and the southwestern region (7.7 μg/m^3^) [40]. It is also possible that the pollutants may be transported from the Pearl River Delta region through the air mass and affect the concentrations of BTEX in the air of Hainan Province [43]. In addition, Sanya City is located in the tropical area, which may accelerate the release of BTEX into the air and increase the concentrations of BTEX. Furthermore, three schools in the study area are located in the city center of Sanya and close to main roads. Vehicle exhaust is another significant source of BTEX exposure for children. Studies have shown that traffic emissions were the primary source of VOCs in Hainan Island [43]. Furthermore, children may also be exposed to BTEX through dietary intake. For example, ethylbenzene also exists in foods like fruits, beans, and honey [44].

Exposure to BTEX can affect human reproductive health. Benzene has been found to affect sperm motility and sperm count in men, as well as disrupt the menstrual cycle and hormones in women and extend the menstrual cycle [10]. Animal studies suggested that xylene may delay the development and maturation of testicular mesenchymal cells and reduce testosterone production in adolescent male rats by inducing the production of reactive oxygen species (ROS) [45]. Exposure to xylene was linked to an evaluated risk of oligomenorrhea in women [46]. A study reported that exposure to toluene affected sex hormone levels in females mediated by serum albumin [47]. Moreover, ethylbenzene exposure can induce autophagy and apoptosis, which prevents follicular cells from growth and development, and subsequently affecting the release of steroid hormones [48]. Limited research has reported the relationship between BTEX exposure and sexual development in children. A previous study found that children living in a suburban area with a waste incinerator had higher concentrations of MU (benzene metabolite) in their urine, reached sexual maturity at an older age and had smaller testicular volume among boys than those in the control area [49]. Similarly, urinary levels of MU were elevated in non-PP children compared to PP children in our study.

An important finding in our study was that 1,2-DB exposure might be a risk factor for PP and EP. In addition, when the parental education level was below high school, PMA was positively associated with EP in children. 1,2-DB, also called catechol, and PMA are metabolites of benzene. Benzene is widely found in solvents and daily products. Benzene, toluene, ethylbenzene, and xylene have been identified as endocrine disrupting chemicals [50]. They may interfere with the hypothalamic–pituitary–gonadal (HPG) axis, reducing ovarian steroid hormone secretion by inhibiting gonadotropin-releasing hormone (GnRH) or pituitary gonadotropin production [51,52]. Benzene may act as an endocrine disruptor, affecting hormone synthesis or interfering with the binding of hormones to their respective receptors, and affecting sex hormone balance [52,53]. Studies have shown correlations between high benzene levels in follicular fluid and elevated FSH levels and reduced estradiol levels in groups of women undergoing in vitro fertilization [54]. We suppose that BTEX exposure, especially benzene exposure has multiple roles in children’s pubertal development and may advance or delay puberty onset. Nevertheless, the levels of sex hormones in children with PP and EP may also affect the metabolism of BTEX in children.

One unexpected finding is that 1,2-DB and MU are both metabolites of benzene, but they were associated with PP in different directions. A possible explanation is that the structural differences between the two metabolites lead to effect differences. 1,2-DB (Catechol) is formed by oxidation of phenol (benzene’s oxidized metabolite) catalyzed by cytochrome P450 2E1 (CYP2E1) enzymes, whereas MU is formed by the oxidative hydrolysis of benzene with ring opening reaction [55]. Benzene exerts its toxic effects through its metabolites. As a phenolic metabolite of benzene, 1,2-DB is readily oxidized to form semiquinone and reactive quinone, which reacts to generate ROS, inducing further oxidative stress, and thus interfering with cellular signaling pathways and altering cellular homeostasis, which in turn triggers toxic effects [10]. A positive correlation between the oxidative DNA damage biomarker 8-OHdG and 1,2-DB was also found in our study. In addition, 1,2-DB are structurally similar to sex hormones. Prior studies have found positive associations between exposure to phenolic compounds and PP risk [56]. We hypothesize that 1,2-DB has endocrine-disrupting properties and may exert estrogenic effects by binding to estrogen receptors, leading to increased endogenous estrogen production [57]. Furthermore, 1,2-DB may act synergistically or antagonistically with its parent compound, benzene. Some studies found inconsistent results that benzene metabolites in the urine of exposed children have been found to correlate to delayed sexual maturity [49]. This might be because benzene delays sexual maturation through its toxic metabolites. However, the metabolite 1,2-DB, due to its phenolic structure (especially the catechol structure), may advance children’s pubertal development. However, there was no significant association between 1,2-DB and PP adjusted for confounders. The possible explanation is that PP is more influenced by other confounders, especially BMI. Previous studies have found that obesity is a key risk factor for PP [58]. BTEX are lipophilic and tend to accumulate in the fat of human body. Studies have shown that urinary concentrations of BTEX metabolites obese population were higher than those of normal-weight population [59]. The impact of obesity on PP may be greater than that of BTEX on it. Furthermore, the quantitative measurement of BTEX metabolites in urine only reflects short-term exposure.

We also found that PGA was negatively associated with EP. Animal studies indicated that inhalation of ethylbenzene did not impact the structure, function, and fertility of the ovaries in rats [60]. Research on the possible endocrine disruption of ethylbenzene is limited. One study showed that mandelic acid, a metabolite of ethylbenzene, was negatively correlated with FSH levels, but another study revealed no significant association among mandelic acid, PGA, and FSH levels [61]. In addition, PGA is also a metabolite of styrene. Recent studies have indicated that styrene lacks endocrine-disrupting potential [62].

The associations between BTEX metabolites and EP varied according to the level of parental education, which may be related to living area, family environment, and dietary and nutritional status. However, benzene exposure has an impact on early puberty regardless of the level of parental education. Therefore, it is necessary to focus on benzene exposure.

Our study is the first to assess the relationship of BTEX exposure with precocious puberty and early puberty, filling a research gap but with some limitations. The concentrations of BTEX metabolites in urine sample were measure once, which only reflects short-term exposures. Longitudinal investigations are required to further validate the findings. In addition, a limited number of confounding variables were included in this study, but other factors also can affect PP in children, such as ambient temperature and sunshine duration. Finally, the associations observed in this study do not indicate causality. The biological mechanisms of BTEX exposure affecting children’s pubertal development need to be further explored.

## 5. Conclusions

Benzene metabolite 1,2-DB was positively associated with PP and EP, especially EP. The association between BTEX exposure and EP varied by parental education level. Attention should be focused on the impact of benzene exposure on children’s pubertal development and action should be implemented to reduce children’s exposure to benzene. The effects of BTEX exposure on PP and EP in children are not associated with oxidative DNA damage, but it is essential to consider the oxidative stress induced by BTEX exposure. Furthermore, further research is needed to investigate the mechanisms by which BTEX exposure influences pubertal development.

## Figures and Tables

**Table 1:** Characteristics of precocious puberty children, early puberty children, and their corresponding controls.

Characteristic	PP (n = 61)	Non-PP (n = 48)	*p*	EP (n = 185)	Non-EP (n = 179)	*p*
Age (years), mean ± SD	6.89 ± 0.61	6.92 ± 0.61	0.790 ^a^	8.36 ± 0.49	8.39 ± 0.50	0.649
BMI (kg/m^2^), mean ± SD	17.7 ± 3.10	16.0 ± 2.88	0.001	17.5 ± 2.97	15.5 ± 2.07	0.000
Sex, n (%)			0.841 ^b^			0.371
Male	13 (21.3)	11 (22.9)		11 (5.9)	7 (3.9)	
Female	48 (78.7)	37 (77.1)		174 (94.1)	172 (96.1)	
Birth weight, n (%)			0.344			0.513
<2500 g	4 (6.6)	7 (14.6)		11 (5.9)	13 (7.3)	
2500–4000 g	52 (85.2)	36 (75.0)		159 (85.9)	146 (81.6)	
≥4000 g	5 (8.2)	5 (10.4)		15 (8.1)	20 (11.2)	
Mode of delivery, n (%)			0.012			0.183
Natural birth	30 (49.2)	35 (72.9)		102 (55.1)	111 (62.0)	
Cesarean section	31 (50.8)	13 (27.1)		83 (44.9)	68 (38.0)	
Feeding style during the first 4 months, n (%)			0.758			0.387
Breastfeeding	39 (63.9)	31 (64.6)		97 (52.4)	102 (57.0)	
Mixed feeding	9 (14.8)	5 (10.4)		35 (18.9)	37 (20.7)	
Artificial feeding	13 (21.3)	12 (25.0)		53 (28.6)	40 (22.3)	
The only child, n (%)			0.024			0.001
No	44 (72.1)	43 (89.6)		118 (63.8)	142 (79.3)	
Yes	17 (27.9)	5 (10.4)		67 (36.2)	37 (20.7)	
Parental education level ^c^, n (%)			0.003			0.675
Below high school	7 (11.5)	17 (35.4)		33 (17.8)	35 (19.6)	
High school or above	54 (88.5)	31 (64.6)		152 (82.2)	144 (80.4)	
Frequency of supplementing vitamin D			0.251			0.002
0	49 (80.3)	37 (77.1)		156 (84.3)	127 (70.9)	
Less than twice a week	9 (14.8)	10 (20.8)		18 (9.7)	41 (22.9)	
3–6 times a week	3 (4.9)	0 (0.0)		10 (5.4)	7 (3.9)	
≥7 times a week	0 (0.0)	1 (2.1)		1 (0.5)	4 (2.2)	

^a^ Mann—Whitney U test was employed to compare continuous variables. ^b^ chi-square test was employed to compare categorical variables. ^c^ When educational level of father or mother was high school and above, it was categorized as high school or above.

**Table 2:** Creatinine-adjusted concentrations of urinary BTEX metabolites and 8-OHdG of children in precocious puberty and non-precocious puberty subgroups (μg/g creatinine).

		Analyte	PMA	1,2-DB	MU	BMA	PGA	2MHA	3MHA	8-OHdG
Total (n = 109)	PP (n = 61)	DF	96.7%	96.7%	100%	96.7%	100%	100%	100%	100%
		GM	7.09	60.1	52.5	3.71	418	51.3	44.9	6.21
		Median	8.69	85.3	47.4	3.74	496	39.3	42.5	5.31
	Non-PP (n = 48)	DF	93.8%	87.5%	100%	95.8%	97.9%	97.9%	100%	100%
		GM	6.56	29.7	67.6	4.12	406	46.8	42.9	5.71
		Median	7.84	42.4	60.0	4.30	468	47.9	39.9	6.02
		*p* ^a^	0.784	0.015	0.046	0.550	0.840	0.705	0.913	0.462
Girl (n = 85)	PP (n = 48)	GM	6.81	55.9	55.2	4.30	428	50.0	44.5	6.19
		Median	8.95	83.6	54.1	3.90	496	40.7	43.6	5.50
	Non-PP (n = 37)	GM	6.76	24.2	71.0	5.54	462	53.4	41.2	4.95
		Median	7.95	35.5	61.2	5.12	455	48.4	35.2	5.57
		*p*	0.958	0.020	0.079	0.300	0.936	0.451	0.703	0.832
Boy (n = 24)	PP (n = 13)	GM	8.22	78.8	43.8	2.15	384	56.2	46.4	6.29
		Median	5.43	88.2	42.0	2.46	379	34.5	28.0	5.26
	Non-PP (n = 11)	GM	5.90	59.1	57.5	1.53	264	30.1	49.5	9.19
		Median	6.52	69.5	53.0	2.42	600	42.3	52.9	9.02
		*p*	0.733	0.424	0.252	0.955	1.000	0.569	0.494	0.134

Abbreviations: PP, precocious puberty; DF, detection frequency; GM, geometric mean; PMA, S-phenyl mercapturic acid; 1,2-DB, 1,2-dihydroxybenzene (catechol); MU, *trans*,*trans*-muconic acid; BMA, N-Acetyl-S-(benzyl)-L-cysteine; PGA, phenylglyoxylic acid; 2MHA, 2-methylhippuric acid; 3MHA, 3-methylhippuric acid. 8-OHdG: 8-hydroxy-2′-deoxyguanosine. ^a^ Mann–Whitney U test.

**Table 3:** Creatinine-adjusted concentrations of urinary BTEX metabolites and 8-OHdG of children in early puberty and non-early puberty subgroups (μg/g creatinine).

		Analyte	PMA	1,2-DB	MU	BMA	PGA	2MHA	3MHA	8-OHdG
Total (n = 364)	EP (n = 185)	DF	97.3%	100%	99.5%	96.8%	98.4%	99.5%	100%	100%
		GM	5.74	75.0	48.2	4.12	284	39.7	37.7	6.10
		Median	6.35	62.0	42.7	4.60	348	36.8	32.8	5.58
	Non-EP (n = 179)	DF	99.4%	96.1%	100%	99.4%	99.4%	100%	99.4%	100%
		GM	5.63	44.3	52.7	4.46	381	45	34.7	6.31
		Median	5.90	48.1	48.5	4.31	462	39.5	32.5	5.90
		*p* ^a^	0.779	0.002	0.386	0.709	0.003	0.107	0.500	0.129
Girl (n = 346)	EP (n = 174)	GM	5.61	75.02	47.2	4.02	285	39.6	37.4	5.95
		Median	6.22	61.3	42.7	4.55	354	37.2	33.2	5.51
	Non-EP (n = 172)	GM	5.71	44.5	53.1	4.45	382	45.1	34.4	6.26
		Median	5.96	48.5	49.2	4.30	462	40.8	32.5	5.87
		*p*	0.979	0.004	0.271	0.822	0.005	0.121	0.442	0.105
Boy (n = 18)	EP (n = 11)	GM	8.38	74.4	66.7	5.88	271	42.1	42.4	8.99
		Median	6.93	80.6	55.7	5.84	286	28.1	26.2	7.48
	Non-EP (n = 7)	GM	3.97	39.9	43.7	4.60	360	42.1	42.2	3.97
		Median	4.89	29.6	35.5	5.65	417	35.6	27.6	4.89
		*p*	0.151	0.246	0.659	0.536	0.479	0.659	0.724	0.930

Abbreviations: EP, early puberty; DF, detection frequency; GM, geometric mean; PMA, S-phenyl mercapturic acid; 1,2-DB, 1,2-dihydroxybenzene (catechol); MU, *trans*,*trans*-muconic acid; BMA, N-Acetyl-S-(benzyl)-L-cysteine; PGA, phenylglyoxylic acid; 2MHA, 2-methylhippuric acid; 3MHA, 3-methylhippuric acid. 8-OHdG: 8-hydroxy-2′-deoxyguanosine. ^a^ Mann–Whitney U test.

**Table 4:** Association of urinary BTEX metabolites and 8-OHdG with early puberty in different parental educational level.

Analyte	Parental Education Level			
	Below High School (n = 68)	*p* ^a^	High School or Above (n = 296)	*p*
PMA	3.66 (1.32, 10.2)	0.013	0.81 (0.61, 1.08)	0.152
1,2-DB	1.55 (0.86, 2.81)	0.147	1.35 (1.06, 1.74)	0.014
MU	0.54 (0.20, 1.44)	0.220	0.92 (0.66, 1.28)	0.626
BMA	0.90 (0.54, 1.50)	0.677	0.98 (0.76, 1.26)	0.867
PGA	0.45 (0.18, 1.16)	0.098	0.74 (0.56, 0.97)	0.032
2MHA	0.98 (0.20, 4.76)	0.983	0.76 (0.46, 1.23)	0.260
3MHA	0.65 (0.17, 2.48)	0.531	1.42 (0.89, 2.26)	0.142
8-OHdG	1.43 (0.27, 7.69)	0.675	0.69 (0.39, 1.24)	0.214

^a^ Model was adjusted for body mass index, mode of delivery, and whether or not only child.

## Data Availability

The data presented in this study are available on request from the corresponding author.

## Supplementary Materials

The following supporting information can be downloaded at: https://www.mdpi.com/article/10.3390/antiox14101164/s1, Text S1: Sample preparation and determination methods of 8-OHdG and creatinine in urine. Table S1: Information of target analytes. Table S2: Chromatographic gradient elution parameters for BTEX metabolites. Table S3: Mass spectrometry parameters of BTEX metabolites, 8-OHdG, and creatinine. Table S4: Recoveries and limits of detection (LODs) in this study. Table S5: Dietary differences between the precocious puberty (PP) and control children. Table S6: Unadjusted concentrations of urinary BTEX metabolites and 8-OHdG of children in the precocious puberty and non-precocious puberty subgroups, as well as the early puberty and non-early puberty subgroups (ng/mL). Table S7: Comparison of urinary concentrations of BTEX metabolites between precocious puberty children and early puberty children (μg/g creatinine). Table S8: Associations between urinary levels of BTEX metabolites and 8-OHdG (ln-transformed) by multiple linear regression. Table S9: Associations of BTEX metabolites and 8-OHdG with precocious puberty using Binary logistic regression. Table S10: The WQS regression model estimated BTEX metabolite mixture associated with precocious puberty and early puberty based on ln-transformed urinary concentrations. Table S11: Odds ratios (ORs) and 95% CI for precocious puberty in girls (n = 85) associated with urinary concentrations of BTEX metabolites and 8-OHdG. Table S12: Associations of urinary BTEX metabolites and 8-OHdG with early puberty using Binary logistic regression. Table S13: Odds ratios (ORs) and 95% CI for early puberty in girls (n = 346) associated with urinary concentrations of BTEX metabolites and 8-OHdG. Table S14: Urinary concentrations of BTEX metabolites in different studies.
