# Supplementary material for: Internal Exposure to BTEX in Tropical Children: Does Exposure Speed Up Pubertal Development?
Source: Antioxidants (Basel). 2025 Sep 25;14(10):1164. doi: 10.3390/antiox14101164 (PMC12561042; doi:10.3390/antiox14101164)
Supplement: Supplementary file 1 [file antioxidants-14-01164-s001.zip › antioxidants-3826407-supplementary.pdf]

Supporting Information

Contents

Supplemental text

Text S1. Sample preparation and determination methods of 8-OHdG and creatinine in urine.

Supplemental tables

Table S1. Information of target analytes.

Table S2. Chromatographic gradient elution parameters for BTEX metabolites.

Table S3. Mass spectrometry parameters of BTEX metabolites, 8-OHdG, and creatinine.

Table S4. Recoveries and limits of detection (LODs) in this study.

Table S5. Dietary differences between the precocious puberty (PP) and control children.

Table S6. Unadjusted concentrations of urinary BTEX metabolites and 8-OHdG of children in the precocious puberty and non-precocious puberty subgroups, as well as the early puberty and non-early puberty subgroups (ng/mL).

Table S7. Comparison of urinary concentrations of BTEX metabolites between precocious puberty children and early puberty children (μg/g creatinine).

Table S8. Associations between urinary levels of BTEX metabolites and 8-OHdG (ln-transformed) by multiple linear regression.

Table S9. Associations of BTEX metabolites and 8-OHdG with precocious puberty using

Binary logistic regression.

Table S10. The WQS regression model estimated BTEX metabolite mixture associated with precocious puberty and early puberty based on ln-transformed urinary concentrations.

Table S11. Odds ratios (ORs) and 95% CI for precocious puberty in girls (n = 85) associated with urinary concentrations of BTEX metabolites and 8-OHdG.

Table S12. Associations of urinary BTEX metabolites and 8-OHdG with early puberty using Binary logistic regression.

Table S13. Odds ratios (ORs) and 95% CI for early puberty in girls (n = 346) associated with urinary concentrations of BTEX metabolites and 8-OHdG.

Table S14. Urinary concentrations of BTEX metabolites in different studies.

References

Text S1. Sample preparation and determination methods of 8-OHdG and creatinine in urine.

8-OHdG standard and method.

8-OHdG was purchased from Sigma-Aldrich and its internal standard $^{15}N_5$-8-OHdG was purchased from Cambridge Isotope Laboratories. The purities are greater than 98%.

900 μL HPLC water, 50 μL urine sample and 50 μL internal standard solution (200 ng/mL) were spiked into amber LC vial in order. The sample mixture was vortexed, filtered through nylon membrane and collected in amber LC vial for further analysis.

8-OHdG was determined by high-performance liquid chromatography tandem triple quadrupole mass spectrometry (HPLC-MS/MS). Betasil C18 column (100 mm×2.1 mm, 5 μm) was used for the chromatographic separation of 8-OHdG. The mobile phases were 0.1% formic acid in water (A) and methanol (B) at a flow rate of 0.4 mL/min with sample injection volume of 5 μL. The elution gradient

was 11%B from 0 to 0.5 min, increased to 80%B from 0.5 min to 1.6min and held for 2.9 min, decreased to 11%B from 4.5 min to 5min and held for 1.5 min to equilibrate the column. The limit of determination (LOD) for 8-OHdG was 0.012 ng/mL.

Creatinine standard and method.

Creatinine was purchased from Sigma-Aldrich. Creatinine-d_3 was purchased from Beijing Manhage Bio-Technology company LTD.

A 20 μL aliquot of urine sample was injected into 10 mL HPLC water in a 15 mL polypropylene centrifuge tube. The sample mixture was vortexed. Then, 900 μL HPLC water, 50 μL of the sample mixture and 50 μL creatinine internal standard solution (10 μg/mL) were spiked into amber LC vial in order, vortexed and analyzed within two days.

Creatinine was determined using HPLC-MS/MS. Betasil C18 column (100 mm×2.1 mm, 5 μm) was used for the chromatographic separation of creatinine. The mobile phase gradient was isocratic with 50% water (A) and 50% methanol (B) for 4 min at a flow rate of 0.4 mL/min. The sample injection volume was 2 μL. The LOD for creatinine was 0.061 ng/mL.

Table S1. Information of target analytes.

Number	Parent compound	Metabolite	Abbreviation	CAS number	Manufacturer, purity	Internal standard	CAS number	Manufacturer, purity
1	Benzene	S-Phenyl mercapturic acid	PMA	4775-80-8	Toronto Research Chemicals (Toronto, Canada), >95%	PMA-d_5	1331906-27-4	Toronto Research Chemicals (Toronto, Canada), >95%
2		1,2-Dihydroxybenzene (Catechol)	1,2-DB	120-80-9	Dr. Ehrenstorfer GmbH, 99.8%	1,2-DB-d_4	103963-58-2	
3		*trans,trans*-Muconic acid	MU	3588-17-8	Sigma-Aldrich, >98%	MU-d_4	1955496-83-9	C/D/N Isotopes (Pointe-Claire, Quebec, Canada), >99.6%
4	Toluene	N-Acetyl-S-(benzyl)-L-cysteine	BMA	19542-77-9	Toronto Research Chemicals (Toronto, Canada), >95%	BMA-d_5	1955496-81-7	Toronto Research Chemicals (Toronto, Canada), >95%
5	Ethylbenzene, styrene	Phenylglyoxylic acid	PGA	611-73-4		PGA-d_5	1217089-53-6	
6	Xylene	2-Methyl hippuric acid	2MHA	42013-20-7		2MHA-d_7	1216430-90-8	
7		3-Methyl hippuric acid	3MHA	27115-49-7		3MHA-d_7	1216551-07-3	

Table S2. Chromatographic gradient elution parameters for BTEX metabolites.

Time (min)	Flow (mL/min)	Solvent A	Solvent B
0	0.4	95%	5%
1	0.4	95%	5%
2	0.4	70%	30%
5	0.4	10%	90%
7.5	0.4	10%	90%
8.5	0.4	95%	5%
11	0.4	95%	5%

Table S3. Mass spectrometry parameters of BTEX metabolites, 8-OHdG, and creatinine.

Analyte	Precursor ion (m/z)	Product ion (m/z)	DP(V)	CE (V)	CXP(V)
PMA	237.9	109.0	−61.3	−40.0	−5.0
PMA-d_5	242.8	113.9	−47.9	−29.2	−9.0
1,2-DB	108.9	91.0	−53.0	−25.0	−20.0
1,2-DB-d_4	112.9	94.0	−51.9	−27.21	−11.72
MU	140.9	53.0	−17.1	−14.0	−6.0
MU-d_4	144.8	57.1	−15.0	−15.7	−7.7
BMA	252.3	123.0	−18.1	−18.1	−6.1
BMA-d_5	257.3	128.0	−21	−19.06	−15.02
PGA	149.1	77.0	−19.8	−18.1	−11.8
PGA-d_5	153.8	82.0	−29.2	−19.9	−12.3
2MHA	191.8	91.0	−39.0	−20.0	−11.1
2MHA-d_7	199.0	98.0	−37.3	−23.2	−15.7
3MHA	192.0	91.0	−28.0	−17.8	−27.9
3MHA-d_7	199.0	98.0	−30.9	−20.1	−27.2
8-OHdG	284.0	168.0	35	23	18.95
$^{15}N_5$-8-OHdG	289.0	173.0	35	23	18.95
Creatinine	114.0	44.0	60	13	12
Creatinine-d_3	117.0	47.0	60	13	12

Table S4. Recoveries and limits of detection (LODs) in this study.

Analyte	Procedural blank	Blank spiked sample	Matrix spiked sample	Urine sample	LOD (ng/mL)	RSD
	Internal recoveries (%)					
PMA-d_5	94 ± 13[a]	101 ± 14	76 ± 16	78 ± 16	-	0%–26%[b]

1,2-DB-d_4	107 ± 22	117 ± 17	64 ± 20	61 ± 21	-	0%–15%
MU-d_4	97 ± 13	101 ± 11	85 ± 15	91 ± 20	-	0%–21%
BMA-d_5	104 ± 9	108 ± 7	89 ± 11	93 ± 13	-	0%–12%
PGA-d_5	102 ± 14	96 ± 14	99 ± 14	114 ± 21	-	0%–8%
2MHA-d_7	100 ± 12	105 ± 12	80 ± 16	82 ± 14	-	0%–20%
3MHA-d_7	95 ± 11	99 ± 13	67 ± 16	72 ± 15	-	0%–15%
	Spiked recoveries (%) (Spiked level: 10 ng/mL)					
PMA	-	108 ± 11	109 ± 23	-	0.05	-
1,2-DB	-	105 ± 9	95 ± 21	-	0.30	-
MU	-	99 ± 7	88 ± 22	-	0.06	-
BMA	-	105±8	96 ± 15	-	0.01	-
PGA	-	101 ± 12	132 ± 49	-	0.49	-
2MHA	-	101 ± 9	89 ± 14	-	0.03	-
3MHA	-	104 ± 9	100 ± 15	-	0.01	-

RSD: relative standard deviation.

[a] Mean ± SD. [b] Minimum to maximum.

Table S5. Dietary differences between the precocious puberty (PP) and control children.

Dietary intake	PP (n = 61)	Control (n = 48)	*p*
Vegetable, n (%)			0.032
0	2 (3.3)	9 (18.8)	
1-3 times/ week	12 (19.7)	7 (14.6)	
≥4 times/ week	47 (77.0)	32 (66.7)	
Fruit, n (%)			0.043
0	3 (4.9)	9 (18.8)	
1-3 times/ week	19 (31.1)	9 (18.8)	
≥4 times/ week	39 (64.0)	30 (62.5)	
Animal colostrum, n (%)			0.212
0	51 (83.6)	44 (91.7)	
1-3 times/ week	10 (16.4)	4 (8.3)	
Milk products, n (%)			0.098
0	5 (8.2)	11 (22.9)	
1-3 times/ week	18 (29.5)	12 (25.0)	
≥4 times/ week	38 (62.3)	25 (52.1)	
Red meat, n (%)			0.070
0	8 (13.1)	15 (31.3)	
1-3 times/ week	14 (23.0)	9 (18.8)	
≥4 times/ week	39 (63.9)	24 (50.0)	
Seafoods, n (%)			0.201

0	11 (18.1)	10 (20.8)	
1-3 times/ week	40 (65.6)	24 (50.0)	
≥4 times/ week	10 (16.4)	14 (29.2)	
Bean products, n (%)			0.665
0	15 (24.6)	13 (27.1)	
1-3 times/ week	36 (59.0)	30 (62.5)	
≥4 times/ week	10 (16.4)	5 (10.4)	

Table S6. Unadjusted concentrations of urinary BTEX metabolites and 8-OHdG of children in the precocious puberty and non-precocious puberty subgroups, as well as the early puberty and non-early puberty subgroups (ng/mL).

Analyte	PP (n = 61)			Control (n = 48)			p^a	EP (n = 185)			Control (n = 179)			p
	DF	GM	Median	DF	GM	Median		DF	GM	Median	DF	GM	Median	
PMA	96.7%	8.75	10.3	93.8%	7.51	9.90	0.735	97.3%	8.32	10.6	99.40%	7.88	9.20	0.190
1,2-DB	96.7%	74.2	86.0	87.5%	34.0	56.9	0.021	100%	109	111	96.10%	62.0	71.0	0.000
MU	100%	64.9	66.5	100%	77.5	91.6	0.203	99.5%	69.9	66.4	100%	73.7	71.0	0.801
BMA	96.7%	4.58	6.02	95.8%	4.73	6.59	0.742	96.8%	5.97	7.01	99.40%	6.23	7.01	0.477
PGA	100%	516	687	97.9%	466	638	0.903	98.4%	412	570	99.40%	533	701	0.091
2MHA	100%	63.3	57.9	97.9%	53.7	61.6	0.781	99.5%	57.6	64.1	100%	62.9	62.1	0.708
3MHA	100%	55.4	55.4	100%	49.2	49.9	0.435	100%	54.6	57.5	99.40%	48.5	50.9	0.116
8-OHdG	100%	7.64	8.37	100%	6.54	7.84	0.718	100%	8.77	9.04	100%	8.76	9.18	0.998

[a] Mann-Whitney U test. PP, precocious puberty; EP: early puberty.

Table S7. Comparison of urinary concentrations of BTEX metabolites between precocious puberty children and early puberty children (μg/g creatinine).

Analyte	PP (n = 61)				EP (n = 185)				p^a
	GM	25th	Median	75th	GM	25th	Median	75th	
PMA	7.09	3.14	8.69	19.4	5.74	3.93	6.35	9.76	0.112
1,2-DB	60.1	40.3	85.3	161	75.0	37.7	62.0	151	0.761
MU	52.5	28.7	47.4	89.2	48.2	25.4	42.7	90.5	0.493
BMA	3.71	2.58	3.74	8.14	4.12	2.81	4.60	8.43	0.362
PGA	418	316	496	628	284	199	348	600	0.021
2MHA	51.3	24.8	39.3	95.1	39.7	24.4	36.8	57.9	0.180
3MHA	44.9	26.8	42.5	59.6	37.7	25.5	32.8	51.6	0.205
8-OHdG	6.21	4.50	5.31	8.11	6.10	4.14	5.58	7.50	0.928

[a] Mann-Whitney U test. PP: precocious puberty; EP: early puberty.

Table S8. Associations between urinary levels of BTEX metabolites and 8-OHdG (ln-transformed) by multiple linear regression.

	Compound	β	95%CI of β	p^a
PP children (n = 61)	PMA	0.04	(−0.07, 0.14)	0.493
	1,2-DB	0.11	(0.02, 0.19)	0.021
	MU	0.12	(−0.08, 0.32)	0.250
	BMA	−0.02	(−0.14, 0.09)	0.682
	PGA	0.12	(−0.05, 0.28)	0.162
	2MHA	−0.21	(−0.48, 0.06)	0.122
	3MHA	0.45	(0.16, 0.74)	0.003
Non-PP children (n = 48)	PMA	−0.06	(−0.38, 0.27)	0.717
	1,2-DB	−0.07	(−0.26,0.13)	0.481
	MU	0.13	(−0.48, 0.74)	0.667
	BMA	0.10	(−0.25, 0.44)	0.573
	PGA	−0.03	(−0.55, 0.50)	0.918
	2MHA	−0.33	(−0.96, 0.30)	0.298
	3MHA	0.29	(−0.24, 0.81)	0.275
EP children (n = 185)	PMA	−0.01	(−0.09, 0.07)	0.791
	1,2-DB	0.14	(0.07, 0.22)	0.000
	MU	0.04	(−0.06, 0.13)	0.464
	BMA	0.01	(−0.05, 0.07)	0.653
	PGA	0.02	(−0.05, 0.09)	0.580
	2MHA	0.12	(−0.02, 0.26)	0.080

	3MHA	0.08	(−0.07, 0.23)	0.291
Non-EP children (n = 179)	PMA	0.01	(−0.06, 0.07)	0.830
	1,2-DB	0.05	(0.01, 0.09)	0.029
	MU	0.05	(−0.03, 0.13)	0.256
	BMA	−0.05	(−0.12, 0.02)	0.168
	PGA	0.06	(−0.01, 0.13)	0.091
	2MHA	0.20	(0.09, 0.32)	0.001
	3MHA	0.06	(−0.04, 0.15)	0.230

[a] Model was adjusted by sex, age, body mass index, mode of delivery, whether or not the only child, and parental education level. PP: precocious puberty; EP: early puberty.

Table S9. Associations of BTEX metabolites and 8-OHdG with precocious puberty using Binary logistic regression.

Compound	Crude OR (95%CI)	*p*	Adjusted OR (95%CI) [a]	*p*
PMA	1.09 (0.81, 1.48)	0.558	1.16 (0.83, 1.62)	0.389
1,2-DB	1.27 (1.01, 1.60)	0.042	1.17 (0.92, 1.50)	0.206
MU	0.49 (0.27, 0.90)	0.021	0.47 (0.24, 0.92)	0.027
BMA	1.00 (0.71, 1.40)	1.000	1.05 (0.75, 1.48)	0.771
PGA	1.01 (0.62, 1.66)	0.961	1.06 (0.62, 1.82)	0.818
2MHA	1.12 (0.60, 2.08)	0.721	1.09 (0.57, 2.09)	0.79
3MHA	1.13 (0.60, 2.13)	0.697	1.03 (0.54, 1.98)	0.925
8-OHdG	1.16 (0.74, 1.82)	0.526	1.17 (0.71, 1.93)	0.529

[a] Model was adjusted for body mass index, mode of delivery, whether or not the only child, and parental education level.

Table S10. The WQS regression model estimated BTEX metabolite mixture associated with precocious puberty and early puberty based on ln-transformed urinary concentrations.

Outcome	OR	95%CI of OR	*p*
Precocious puberty			
Positive	1.56	(0.76, 3.19)	0.223
Negative	0.96	(0.47, 1.95)	0.903
Early puberty			
Positive	1.26	(0.87, 1.82)	0.225
Negative	0.51	(0.34, 0.76)	0.001

[a] Models were adjusted for body mass index, mode of delivery, whether or not the only child, and parental education level.

Table S11. Odds ratios (ORs) and 95% CI for precocious puberty in girls (n = 85) associated with urinary concentrations of BTEX metabolites and 8-OHdG.

ompound	Crude OR (95%CI)	*p*	Adjusted OR (95%CI) [a]	*p*
PMA	1.28 (0.88, 1.85)	0.197	1.39 (0.90, 2.13)	0.134
1,2-DB	1.32 (1.02, 1.70)	0.035	1.14 (0.86, 1.52)	0.350
MU	0.42 (0.20, 0.88)	0.022	0.47 (0.20, 1.09)	0.078
BMA	0.73 (0.42, 1.28)	0.278	0.74 (0.42, 1.32)	0.315
PGA	1.04 (0.56, 1.94)	0.896	0.98 (0.48, 2.00)	0.953
2MHA	0.40 (0.13, 1.16)	0.091	0.39 (0.11, 1.35)	0.137
3MHA	3.06 (1.02, 9.15)	0.046	2.10 (0.62, 7.08)	0.231
8-OHdG	1.41 (0.78, 2.54)	0.257	2.06 (0.57, 7.43)	0.270

[a] Model was adjusted for body mass index, mode of delivery, whether or not an only child, and parental education level.

Table S12. Associations of urinary BTEX metabolites and 8-OHdG with early puberty using Binary logistic regression.

Compound	Crude OR (95%CI)	*p*	Adjusted OR (95%CI) [a]	*p*
PMA	1.06 (0.85, 1.32)	0.600	1.02 (0.80, 1.30)	0.857
1,2-DB	1.35 (1.11, 1.63)	0.002	1.36 (1.10, 1.69)	0.004
MU	0.88 (0.68, 1.15)	0.346	0.86 (0.64, 1.16)	0.337
BMA	0.94 (0.77, 1.15)	0.541	0.95 (0.77, 1.18)	0.670
PGA	0.80 (0.63, 1.02)	0.071	0.73 (0.56, 0.93)	0.012
2MHA	0.71 (0.47, 1.10)	0.099	0.74 (0.47, 1.16)	0.192
3MHA	1.61 (1.04, 2.50)	0.034	1.39 (0.90, 2.16)	0.140
8-OHdG	0.77 (0.49, 1.23)	0.278	0.81 (0.48, 1.37)	0.431

[a] Model was adjusted for body mass index, mode of delivery, whether or not the only child, and parental education level.

Table S13. Odds ratios (ORs) and 95% CI for early puberty in girls (n = 346) associated with urinary concentrations of BTEX metabolites and 8-OHdG.

Compound	Crude OR (95%CI)	*p*	Adjusted OR (95%CI) [a]	*p*
PMA	1.03 (0.82, 1.29)	0.787	0.99 (0.78, 1.26)	0.938
1,2-DB	1.35 (1.11, 1.64)	0.003	1.34 (1.08, 1.66)	0.008
MU	0.87 (0.66, 1.14)	0.302	0.86 (0.64, 1.16)	0.326
BMA	0.93 (0.76, 1.14)	0.492	0.95 (0.77, 1.18)	0.662
PGA	0.80 (0.63, 1.03)	0.082	0.74 (0.57, 0.96)	0.023
2MHA	0.74 (0.49, 1.11)	0.145	0.76 (0.48, 1.20)	0.234

3MHA	1.65 (1.05, 2.59)	0.031	1.41 (0.90, 2.21)	0.134
8-OHdG	0.71 (0.44, 1.15)	0.160	0.82 (0.48, 1.40)	0.464

[a] Model was adjusted for body mass index, mode of delivery, whether or not an only child, and parental education level.

Table S14. Urinary concentrations of BTEX metabolites in different studies.

Region	Population	Concentration	PMA	1,2-DB	MU	BMA	PGA	2MHA	3MHA (+4MHA)	Reference
This study	Precocious puberty children	GM (μg/g creatinine)	7.09	60.1	52.5	3.71	418	51.3	44.9	-
	Early puberty children		5.74	75.0	48.2	4.12	284	39.7	37.7	-
Italy	Children in urban area	GM (μg/g creatinine)	0.27	-	111	-	-	-	-	[1]
	Children in rural area		0.22	-	64.2	-	-	-	-	
New York, USA	Pregnant women	Median (ng/mL)	0.642	-	245	5.62	208	21.2	150	[2]
USA	Children aged 6–11 years	GM (ng/mg creatinine)	-	-	115	8.8	231	31.2	217.7	[3]
USA	Infants in incubators	GM (ng/mL)	-	-	-	104	218	-	38.8	[4]
	Infants in open cribs		-	-	-	62.6	28.0	-	23.7	
Northeastern British Columbia, Canada	Pregnant women	Median (μg/g creatinine)	0.18	-	180	7.00	-	-	-	[5]
Guangzhou, China	6–12-year-old children	GM (μg/L)	0.118	986	20.8	4.46	36.2	16.1	90.4	[6]
USA	Healthy participants aged 21–45 years (Nonsmokers)	Mean (ng/mg creatinine)	-	-	139	10.6	186	8.60	292	[7]
South and central	0–7-year-old	SG-adjusted GM	<MDL	-	54.8	3.11	93.9	23.7	54.5	[8]

China	children	(μg/L)								
Southern China	Children	GM (μg/g creatinine)	0.124	7.39	61.6	5.35	-	-	-	[9]
	Adult		0.156	5.36	37.9	3.89	-	-	-	
New York, USA	Healthy volunteers (spot urine)	Creatinine-adjusted GM (μg/g)	<LOD	-	126	2.70	22.2	8.20	53.5	[10]
Korea	general population in KoNEHS Cycle 3	Median (μg/L)	-	-	88.5	4.74	-	-	-	[11]
Southwest China	Nonoccupational populations in R1	GM (μg/g creatinine)	4.23	91.4	213	7.98	-	-	-	[12]
	R2		2.35	58.0	128	4.70	-	-	-	
	R3		2.30	62.8	83.0	6.90	-	-	-	
	R4		2.06	43.0	98.1	7.60	-	-	-	
China	General population in urban areas	SG-adjusted GM (μg/L)	-	-	48.2	-	-	31.8	79.4	[13]

Abbreviation: GM, geometric mean; SG, specific gravity; MDL, method detection limit; LOD, limit of detection.

References:

1. Protano, C.; Guidotti, M.; Manini, P.; Petyx, M.; La Torre, G.; Vitali, M. Benzene exposure in childhood: Role of living environments and assessment of available tools. *Environ. Int.* **2010,** *36* (7), 779–787.
2. Boyle, E. B.; Viet, S. M.; Wright, D. J.; Merrill, L. S.; Alwis, K. U.; Blount, B. C.; Mortensen, M. E.; Moye, J., Jr.; Dellarco, M. Assessment of exposure to VOCs among pregnant women in the national children's study. *Int. J. Env. Res. Public Health* **2016,** *13* (4), 376.
3. Jain, R. B. Levels of selected urinary metabolites of volatile organic compounds among children aged 6–11 years. *Environ. Res.* **2015,** *142*, 461–470.
4. El-Metwally, D.; Chain, K.; Stefanak, M. P.; Alwis, U.; Blount, B. C.; LaKind, J. S.; Bearer, C. F. Urinary metabolites of volatile organic compounds of infants in the neonatal intensive care unit. *Pediatr. Res.* **2018,** *83* (6), 1158–1164.
5. Caron-Beaudoin, E.; Valter, N.; Chevrier, J.; Ayotte, P.; Frohlich, K.; Verner, M.-A. Gestational exposure to volatile organic compounds (VOCs) in Northeastern British Columbia, Canada: A pilot study. *Environ. Int.* **2018,** *110*, 131–138.
6. Kuang, H.; Li, Y.; Jiang, W.; Wu, P.; Tan, J.; Zhang, H.; Pang, Q.; Ma, S.; An, T.; Fan, R. Simultaneous determination of urinary 31 metabolites of VOCs, 8-hydroxy-2′-deoxyguanosine, and trans-3′-hydroxycotinine by UPLC-MS/MS: ^{13}C- and ^{15}N-labeled isotoped internal standards are more effective on reduction of matrix effect. *Anal. Bioanal. Chem.* **2019,** *411* (29), 7841–7855.
7. Keith, R. J.; Fetterman, J. L.; Orimoloye, O. A.; Dardari, Z.; Lorkiewicz, P. K.; Hamburg, N. M.; DeFilippis, A. P.; Blaha, M. J.; Bhatnagar, A. Characterization of volatile organic compound metabolites in cigarette smokers, electronic nicotine device users, dual users, and nonusers of tobacco. *Nicotine Tobacco Res.* **2020,** *22* (2), 264–272.
8. Song, W.; Han, Q.; Wan, Y.; Qian, X.; Wei, M.; Jiang, Y.; Wang, Q. Repeated measurements of 21 urinary metabolites of volatile organic compounds and their associations with three selected oxidative stress biomarkers in 0-7-year-old healthy children from south and central China. *Chemosphere* **2022,** *287*, 132065.
9. Kuang, H.; Li, Y.; Li, L.; Ma, S.; An, T.; Fan, R. Four-year population exposure study: Implications for the effectiveness of e-waste control and biomarkers of e-waste pollution. *Sci. Total Environ.* **2022,** *842*, 156595.
10. Pal, V. K.; Li, A. J.; Zhu, H.; Kannan, K. Diurnal variability in urinary volatile organic compound metabolites and its association with oxidative stress biomarkers. *Sci. Total Environ.* **2022,** *818*, 151704.
11. Lee, I.; Park, H.; Kim, M. J.; Kim, S.; Choi, S.; Park, J.; Cho, Y. H.; Hong, S.; Yoo, J.; Cheon, G. J.; Choi, K.; Park, Y. J.; Moon, M. K. Exposure to polycyclic aromatic hydrocarbons and volatile organic compounds is associated with a risk of obesity and diabetes mellitus among Korean adults: Korean National Environmental Health Survey (KoNEHS) 2015–2017. *Int. J. Hyg. Environ. Health* **2022,** *240*, 113886.
12. Qin, N.; Zhu, Y.; Zhong, Y.; Tian, J.; Li, J.; Chen, L.; Fan, R.; Wei, F. External exposure to btex, internal biomarker response, and health risk assessment of nonoccupational populations near a coking plant in southwest China. *Int. J. Env. Res. Public Health* **2022,** *19* (2), 847.
13. Yan, M.; Zhu, H.; Luo, H.; Zhang, T.; Sun, H.; Kannan, K. Daily exposure to environmental volatile organic compounds triggers oxidative damage: evidence from a large-scale survey in China. *Environ. Sci. Technol.* **2023,** *57* (49), 20501–20509.
